# Associated factors and cultivation strategies of positive social mentality among college students in the new era

**DOI:** 10.3389/fpsyg.2026.1851175

**Published:** 2026-10-13

**Authors:** Nanqiu Peng, Xiaoli Lv, Li He, Fenghua Zhang

**Affiliations:** 1 College of Medical Technology, Shanghai University of Medicine & Health Sciences, Shanghai, China; 2 Shanghai University of Medicine & Health Sciences Affiliated Zhoupu Hospital, Shanghai, China; 3 Laboratory Quality Management Section, Xuhui District Center for Disease Control and Prevention (Xuhui District Health Supervision Institute), Shanghai, China

**Keywords:** academic year trends, associated factors, cultivation strategies, gender differences, medical students, positive social mentality

## Abstract

**Background:**

In the context of the new era, college students’ positive social mentality plays a pivotal role in their personal development and social harmony.

**Objective:**

This study aimed to examine the current status and correlates of positive social mentality among medical students, with a focus on gender, academic year, and socio-contextual factors.

**Methods:**

A cross-sectional survey was conducted with 123 undergraduate medical laboratory students using a self-developed questionnaire with strong psychometric properties (Cronbach’s α = 0.968; KMO = 0.883). The instrument assessed eight dimensions of positive social mentality (optimism, psychological resilience, self-efficacy, social responsibility, growth mindset, sense of belonging, value identity, and emotional regulation) and six categories of associated factors (family, school, online ecology, peer relationships, employment pressure, and policy perception). Data were analyzed using t-tests, ANOVA, and standardized multiple regression.

**Results:**

No statistically significant gender difference was found in the overall index or in any of the eight dimensions (all *p* ≥ 0.208), and no dimension differed significantly across academic years (all *p* ≥ 0.127); on average value identity scored highest and growth mindset lowest. Standardized multiple regression revealed that school education was by far the strongest positive predictor (β = 0.68), followed by peer relationships (β = 0.18), family factors (β = 0.17) and policy perception (β = 0.16), while online ecology was negatively associated (β = −0.14) and employment pressure was not a significant predictor (β = −0.09, *p* = 0.140); the six factors together explained 81% of the variance (R^2^ = 0.81).

**Conclusion:**

Positive social mentality among medical students is associated with multiple interrelated environmental and relational factors rather than with gender or academic year. Targeted, multi-level interventions addressing these factors are needed to foster students’ psychological well-being and civic engagement.

## 1 Introduction

In the context of the new era—defined as the period since the 19th National Congress of the Communist Party of China (2017), characterized by rapid technological advancement, social transformation, and a focus on high-quality development—college students serve as the driving force of national development. As future builders and successors of the country, their social mentality not only affects their personal growth and academic success but also directly relates to social harmony, stability, and long-term development. The theoretical foundations on which this concept is built are set out below, after which the construct is defined. A positive social mentality enhances students’ ability to integrate into society and reinforces their sense of responsibility and mission, injecting vitality into national development. This proposition is grounded in prior research among Chinese university students, which shows that prosocial dispositions and a sense of social responsibility are closely associated with constructive social engagement and with social adaptation (Xue et al., 2025; Yu et al., 2022). Investigating the associated factors of students’ positive social mentality and exploring effective cultivation strategies thus carries significant theoretical and practical relevance.

Amid ongoing economic transformation and educational reform, the social mentality of college students has undergone substantial changes. While they are presented with unprecedented opportunities, they also face challenges such as employment pressure, academic stress, and complex interpersonal relationships. At the same time, they are actively exploring personal growth and seeking self-actualization. In this evolving landscape, guiding students to develop a positive social mentality has emerged as a crucial task for both educational institutions and broader society. Theoretically, this study draws upon multiple established frameworks. From positive psychology (Fredrickson, 2001; Seligman and Csikszentmihalyi, 2000), we adopt the PERMA model (Positive Emotions, Engagement, Relationships, Meaning, and Accomplishment) to conceptualize the multidimensional nature of psychological well-being. From Bandura’s self-efficacy theory (1977, 1997) (Bandura, 1977; Bandura, 1997), we incorporate the concept of mastery experiences as key drivers of confidence development. Additionally, Bronfenbrenner’s ecological systems theory (1979) provides a framework for understanding how multiple environmental layers—from family (microsystem) to policy (exosystem)—interact to shape students’ social mentality (Bronfenbrenner, 1979). Social mentality theory (Wang, 2020; Wang and Yang, 2018) further informs our understanding of how collective social cognition emerges from individual psychological processes within specific socio-cultural contexts. Drawing on these theoretical foundations, positive social mentality is defined in this study as “a relatively stable psychological tendency characterized by optimistic cognition, positive emotional experiences, and prosocial behavioral intentions toward society, self, and the future, encompassing dimensions such as optimism, resilience, self-efficacy, and social responsibility.” Throughout this article the term “positive social mentality” refers specifically to this multidimensional positive construct rather than to social mentality in general, and the two terms are not used interchangeably; the object of the present study is college students’ positive social mentality.

However, despite growing interest in this topic, several research gaps remain. First, limited empirical research has specifically examined social mentality among medical students, a population facing unique stressors including heavy academic loads, clinical training demands, and direct exposure to human suffering. Second, most existing studies focus on single dimensions (e.g., resilience or optimism) rather than providing a comprehensive, multi-dimensional assessment that captures the complexity of social mentality. Third, grade-level trajectories of social mentality have received insufficient attention, particularly the potential decline during mid-education years. Fourth, few studies have simultaneously examined both individual-level (gender, grade, socioeconomic status) and environmental-level (family, school, online ecology, peer relationships) predictors within a unified analytical framework (Keum and Miller, 2020; Runyon et al., 2024; Zhu et al., 2022). This study aims to explore the status and associated factors of college students’ positive social mentality through a comprehensive questionnaire covering basic demographic variables (e.g., gender, year of study, place of origin) and key dimensions such as confidence in the future, resilience, and social participation awareness. A total of 123 valid responses were collected, offering empirical insight into students’ psychological states and sense of social responsibility. The questionnaire was carefully designed to account for the multifaceted nature of social mentality and included targeted items for each dimension.

Drawing from positive psychology, the cultivation of students’ mental health and positive social mentality has become a central concern for higher education (Chen, 2023; Lian, 2023; Zhang, 2023). Research has shown that fostering such mentality helps shape traits like optimism, resilience, and confidence, thereby improving psychological well-being and social adaptability. Achieving this requires concerted efforts from families, schools, and communities—such as promoting a healthy campus culture, strengthening psychological education, and instilling core socialist values (a set of officially promoted guiding principles in China—prosperity, democracy, civility, harmony, freedom, equality, justice, rule of law, patriotism, dedication, integrity, and friendship) (Bielefeldt and Canney, 2016; Cao, 2024; Jiang et al., 2023).

However, the rapidly evolving social context and rise of the digital age (encompassing social media platforms, educational digital resources, information dissemination channels, and virtual social networks) pose significant challenges to students’ social mentality (Liu et al., 2022). Alongside these digital-related pressures, more traditional stressors such as academic pressure, employment competition, and interpersonal strain can also negatively affect mental health (Barbayannis et al., 2022; Peng et al., 2023). It is thus essential to examine current trends in college students’ positive social mentality and develop effective strategies for intervention (Abas et al., 2023; Bulgarelli et al., 2023; Reeve et al., 2017).

In summary, cultivating college students’ positive social mentality is not only critical to individual growth but also to societal advancement. This study seeks to identify key associated factors and propose actionable strategies to promote mental well-being and civic engagement among college students in the context of the new era. The primary objective of the study is to identify the factors associated with college students’ positive social mentality; developing the questionnaire was a secondary, instrumental aim, undertaken because no existing instrument covers this composite construct, and it is not intended to establish a competing measure of optimism, resilience, self-efficacy or the other dimensions.

## 2 Participants and method

### 2.1 Participants

From April to May 2025, a questionnaire survey was conducted among undergraduate students majoring in medical laboratory science at a medical college in Shanghai. A total of 123 valid responses were collected through the online platform Wenjuanxing. The total number of medical laboratory science students at the participating institution is 480; the survey was distributed to all 480 students, yielding a response rate of 25.6%. The sampling frame therefore comprised the entire accessible population (*N* = 480) and a total-population (census) sampling strategy was used rather than probability sampling from a larger known population. The number of analysable responses was determined by the number of completed questionnaires returned by the invited students (123 of 480; 25.6%) rather than by a pre-specified sample-size target, so no *a priori* power calculation was used to fix the sample size. To establish what the achieved sample could detect, *post hoc* sensitivity analyses were conducted at a significance level of 0.05 (two-tailed) and 80% power. With *N* = 123 the smallest effects detectable were: a zero-order correlation of r ≥ 0.249; a standardized mean difference of d ≥ 0.62 for the male (*n* = 26) versus female (*n* = 97) comparison; a model R^2^ ≥ 0.11 (f^2^ ≥ 0.12) for the six-predictor regression; and η^2^ ≥ 0.08 for the four-group comparison across academic years. Associations smaller than these thresholds cannot be reliably detected with the achieved sample, and the findings reported below should be read in the light of this sensitivity analysis. Among the respondents, 26 were male (21.14%) and 97 were female (78.86%). The participants were distributed across all four academic years: 41 first-year students (33.33%), 25 s-year students (20.33%), 39 third-year students (31.71%), and 18 fourth-year students (14.63%). All 123 participants were of Han ethnicity, which is consistent with the predominant ethnic composition in Shanghai (approximately 91% Han). The study is culturally grounded in the Chinese socio-cultural context: the construct and its associated-factor categories were operationalized with reference to the values and institutional environment emphasized in China’s new era, rather than through ethnicity-specific or indigenous categories. The absence of ethnic variation in this sample is a descriptive feature of the local student population and was not a sampling criterion; consequently no inter-ethnic comparison is possible (see also Section 4.7).

### 2.2 Questionnaire

#### 2.2.1 Questionnaire development and validation

The questionnaire was developed through a systematic process to ensure content validity and psychometric rigor.

Item generation: Initial items were generated based on an extensive literature review of existing social mentality and psychological well-being scales, including the Life Orientation Test-Revised (LOT-R; Scheier et al., 1994), the Connor-Davidson Resilience Scale 10-item version (CD-RISC 10; Connor and Davidson, 2003), and the General Self-Efficacy Scale (GSES; Zhang and Schwarzer, 1995). Items were adapted to the college student context and specifically tailored to capture the eight dimensions of social mentality.

Expert review: Five experts in positive psychology and educational psychology evaluated item relevance, clarity, and representativeness. Items with a Content Validity Index (CVI) below 0.78 were revised or removed, resulting in a final set of 61 items.

Pilot testing: A pilot study was conducted with 30 medical students who were excluded from the final sample. Based on pilot feedback, ambiguous items were reworded, and four reverse-scored items were retained to control for response bias.

The questionnaire consisted of 61 items, structured to gather comprehensive data. It began with 6 demographic items (gender, academic year, place of origin, political affiliation, family economic status, and ethnicity), capturing details such as gender, academic year, and place of origin. The core of the survey included 32 items, which were distributed across eight dimensions of social mentality—optimism, psychological resilience, self-efficacy, social responsibility, growth mindset, sense of belonging, value identity, and emotional regulation—with four items dedicated to each dimension. Additionally, 24 items explored six categories of influencing factors: family background, school education, online environment, peer relationships, employment pressure, and policy perception, also with four items per category. This design ensured a balanced and thorough assessment of both the respondents’ social mentality and the factors that might shape it.

Rationale for the six categories of associated factors. Each category was included because it represents a distinct layer of influence on students’ social mentality and because the corresponding literature identifies it as consequential for this population. Family factors represent the microsystem in which values and expectations are first transmitted, and were measured because family economic stress and family conflict are salient correlates of students’ psychological state. School education represents the mesosystem; ideological and political education, counselors’ support and campus culture were measured because institutional value guidance has been shown to shape students’ social-responsibility attitudes (Bielefeldt and Canney, 2016). Online ecology was measured because social-media use and online information exposure are associated with mental-health outcomes and with social trust (Jiang et al., 2023; Zhu et al., 2022). Peer relationships were included because peer support and peer competition are proximal processes that buffer or aggravate stress during higher education (Keum and Miller, 2020). Employment pressure was measured because labor-market competition and internship experience are salient determinants of students’ confidence in the future. Policy perception was included as the exosystem layer because public policies—such as rural revitalization, the “Double Reduction” policy and social-security provision—shape students’ perceived social equity and life-related anxiety. Together these six categories reflect Bronfenbrenner’s nested environmental layers (Bronfenbrenner, 1979) and the socio-contextual factors identified in prior work on social mentality (Chen, 2023; Lian, 2023; Wang, 2020; Wang and Yang, 2018; Zhang, 2023).

#### 2.2.2 Scoring

Scoring was based on a five-point Likert scale. For positively worded items, scores ranged from 1 (“strongly disagree”) to 5 (“strongly agree”). Items 13, 25, 51 and 56 were reverse-coded (6 minus the original score); no other item was. Within the associated-factor categories several items are negatively worded — family 39 and 41, online ecology 46, 47 and 49, peer relationships 51 and 52, employment pressure 54 and 56 — and these were scored in the direction in which they were presented, so that positively and negatively worded items contribute to the same composite in four of the six categories. The reviewer’s observation on this point is correct, and it is now stated explicitly rather than left implicit. The complete keying scheme is: social-mentality dimensions — items 6–12, 14–24 and 26–37 positively keyed, items 13 and 25 reverse-coded; family factors — items 38 and 40 positively keyed, items 39 and 41 scored as presented; school education — items 42–45 positively keyed; online ecology — item 48 positively keyed, items 46, 47 and 49 scored as presented; peer relationships — items 50 and 53 positively keyed, item 52 scored as presented, item 51 reverse-coded; employment pressure — items 55 and 57 positively keyed, item 54 scored as presented, item 56 reverse-coded; policy perception — items 58–61 positively keyed. The consequences of this keying for the interpretation of the four affected composites are addressed in Section 4.7. Each dimension score was computed as the sum of its four constituent items, and the overall social mentality index was computed as the unweighted mean of the eight dimension scores (equivalently, the sum of all 32 dimension items divided by 8).

#### 2.2.3 Psychometric properties

Reliability analysis indicated excellent internal consistency for the overall questionnaire (Cronbach’s α = 0.968). Validity analysis yielded a KMO value of 0.883, indicating suitability for factor analysis.

Confirmatory factor analysis (CFA) confirmed the eight-factor structure with acceptable model fit: CFI = 0.921, RMSEA = 0.058, SRMR = 0.061, χ^2^/df = 2.14, TLI = 0.907. The Average Variance Extracted (AVE) for each dimension ranged from 0.60 to 0.77, and the square root of AVE for each dimension exceeded inter-construct correlations, supporting discriminant validity (Fornell and Larcker, 1981).

### 2.3 Measurement instruments referenced

The following established instruments were referenced during questionnaire development:

GSES (General Self-Efficacy Scale): The Chinese version (Zhang and Schwarzer, 1995) is a 10-item scale assessing general self-efficacy. It informed the self-efficacy dimension of our questionnaire.
LOT-R (Life Orientation Test-Revised): Scheier et al.’s (1994)10-item measure assessing dispositional optimism was used to guide the development of our optimism dimension.
CD-RISC 10 (Connor-Davidson Resilience Scale 10-item version): The 10-item brief version (Campbell-Sills and Stein, 2007) was referenced for our psychological resilience dimension items.

Rationale for developing a new instrument. Existing measures address single, narrowly defined constructs: the LOT-R assesses dispositional optimism, the CD-RISC 10 assesses psychological resilience, and the GSES assesses general self-efficacy. None of them operationalizes the broader, multidimensional construct of positive social mentality as defined in this study, which additionally encompasses social responsibility, growth mindset, sense of belonging, value identity and emotional regulation, so that no combination of these scales yields the eight-dimension composite required here. These instruments were also developed in other cultural and educational settings and do not capture the socio-contextual conditions of Chinese new-era higher education, nor do they measure the six categories of associated factors (family, school, online ecology, peer relationships, employment pressure and policy perception) that are the focus of the present research question. The instrument developed for this study is therefore not a competing measure: the three established scales were used only as item sources for the corresponding dimensions, and a new instrument was required precisely because the limitations above make existing scales inadequate for the phenomenon under investigation.

### 2.4 Data analysis

Data were analyzed using SPSS 25.0 and AMOS 24.0. Independent-sample t-tests were conducted to compare gender differences in the overall social mentality index and each sub-dimension. One-way analysis of variance (ANOVA) was used to examine differences across academic years. Post-hoc comparisons were conducted using LSD tests. Standardized multiple regression on the observed composite scores was used to examine the simultaneous effects of all six influencing factors on positive social mentality; the coefficients in Table S2 are therefore standardized regression coefficients estimated on manifest composite scores rather than structural-equation-model path estimates from a latent-variable measurement model, a distinction taken up in Section 4.7. A significance level of *p* < 0.05 was used to determine statistical significance. In addition to the six direct paths, an exploratory mediation model was specified in which peer relationships—already one of the six associated-factor categories—were also examined as a proximal pathway linking school education to social mentality. Peer relationships were modeled simultaneously as a direct predictor and as a mediator because peer interaction is both a source of support in its own right and the proximal process through which school-level value guidance and campus culture are transmitted; this dual role follows from Bronfenbrenner’s ecological systems theory (Bronfenbrenner, 1979) and from prior work on peer relationships in student development (Keum and Miller, 2020). The mediation specification is exploratory and is reported as such in Section 3.7.

## 3 Results

### 3.1 Psychometric properties and factor structure

Confirmatory factor analysis (CFA) confirmed the eight-factor structure with acceptable model fit: CFI = 0.921, RMSEA = 0.058, SRMR = 0.061, χ^2^/df = 2.14, TLI = 0.907. The Average Variance Extracted (AVE) for each dimension ranged from 0.60 to 0.77 (see Supplementary Table S1), and the square root of AVE for each dimension exceeded inter-construct correlations, supporting discriminant validity. For example, self-efficacy (AVE = 0.77, √AVE = 0.88) and growth mindset (AVE = 0.65, √AVE = 0.81) showed distinct constructs (inter-correlation = 0.58).

### 3.2 Social mentality scores by dimension

Analysis of the eight dimensions of social mentality revealed that value identity had the highest average score (17.10 ± 2.89), followed by optimism (15.81 ± 3.25) and sense of belonging (15.68 ± 3.08), while growth mindset (13.89 ± 2.10) and psychological resilience (13.93 ± 1.89) had the lowest (Table 1). This suggests that students exhibited the strongest alignment with core values, whereas beliefs about the malleability of ability and recovery from setbacks were relatively weaker.

**Table 1:** Distribution of scores across social mentality dimensions (*N* = 123).

Dimension	Mean	SD	Min	Q1	Median	Q3	Max
Optimism	15.81	3.25	4	14	16	18	20
Psychological resilience	13.93	1.89	8	13	14	16	18
Self-efficacy	15.51	3.16	4	13	16	18	20
Social responsibility	15.59	3.28	4	13	16	18	20
Growth mindset	13.89	2.10	4	13	14	16	20
Sense of belonging	15.68	3.08	4	14	16	18	20
Value identity	17.10	2.89	4	16	17	20	20
Emotional regulation	15.17	3.49	4	13	16	18	20

### 3.3 Gender differences in social mentality

Comparisons between male and female students showed no statistically significant gender difference in the overall index or in any of the eight dimensions (all *p* ≥ 0.208). The largest numerical gap was observed in value identity, where female students scored 0.81 points higher on average than male students (t = −1.27, *p* = 0.208, d = 0.28); every other dimension differed by less than one third of a standard deviation (Table 2).

**Table 2:** Gender differences in social mentality dimensions (*N* = 123).

Dimension	Male (M ± SD)	Female (M ± SD)	*t*	*p*	Cohen’s *d*
Overall index	15.28 ± 2.44	15.35 ± 2.51	−0.13	0.893	0.03
Optimism	15.50 ± 2.96	15.90 ± 3.33	−0.55	0.582	0.12
Psychological resilience	14.15 ± 2.05	13.88 ± 1.85	0.66	0.508	0.15
Self-efficacy	15.62 ± 3.24	15.48 ± 3.15	0.19	0.852	0.04
Social responsibility	15.35 ± 3.24	15.66 ± 3.30	−0.43	0.667	0.10
Growth mindset	14.15 ± 2.31	13.82 ± 2.05	0.71	0.480	0.16
Sense of belonging	15.62 ± 2.67	15.70 ± 3.19	−0.13	0.900	0.03
Value identity	16.46 ± 3.01	17.27 ± 2.85	−1.27	0.208	0.28
Emotional regulation	15.38 ± 3.35	15.11 ± 3.54	0.35	0.726	0.08

### 3.4 Grade-level differences in social mentality

No statistically significant difference across academic years was observed for the overall index or for any of the eight dimensions (all *p* ≥ 0.127). First-year students recorded the numerically highest score on the overall index (15.94 ± 2.16) and the third-year group the lowest (15.16 ± 3.11), and the largest between-year effect was seen for sense of belonging (*F* = 1.94, *p* = 0.127, η^2^ = 0.047); none of these differences reached the conventional significance threshold (Table 3). Because the study was powered only for medium-to-large between-group effects (η^2^ ≥ 0.08; Section 2.1), these null results should be read as inconclusive rather than as evidence of no difference.

**Table 3:** Differences in social mentality dimensions by grade level (*N* = 123).

Dimension	Year 1	Year 2	Year 3	Year 4	*F*	*p*	η^2^
Overall index	15.94 ± 2.16	14.82 ± 1.83	15.16 ± 3.11	15.05 ± 2.35	1.32	0.271	0.032
Optimism	16.29 ± 3.18	15.48 ± 2.18	15.74 ± 3.81	15.33 ± 3.41	0.52	0.671	0.013
Psychological resilience	14.15 ± 1.96	13.48 ± 1.42	14.28 ± 2.05	13.33 ± 1.81	1.73	0.164	0.042
Self-efficacy	16.10 ± 2.96	14.84 ± 2.43	15.46 ± 3.84	15.22 ± 2.84	0.90	0.444	0.022
Social responsibility	16.24 ± 3.23	15.24 ± 2.57	15.15 ± 3.88	15.56 ± 2.81	0.87	0.461	0.021
Growth mindset	14.34 ± 1.39	13.44 ± 1.29	13.79 ± 3.01	13.72 ± 1.90	1.08	0.359	0.027
Sense of belonging	16.59 ± 2.64	15.32 ± 2.59	15.03 ± 3.77	15.56 ± 2.71	1.94	0.127	0.047
Value identity	17.90 ± 2.23	16.52 ± 2.58	16.90 ± 3.59	16.50 ± 2.73	1.74	0.162	0.042
Emotional regulation	15.93 ± 2.80	14.28 ± 3.01	14.95 ± 4.37	15.17 ± 3.29	1.24	0.297	0.030

### 3.5 Influencing factors: dimensional scores and gender differences

The scores for the six categories of influencing factors revealed varying degrees of impact on students’ psychological states. School Education had the highest average score (15.60 ± 3.46 on the 4–20 composite scale), followed by Policy Perception (14.79 ± 3.09) and Peer Relationships (14.07 ± 1.82), whereas Employment Pressure (12.91 ± 2.38) and Online Ecology (13.24 ± 3.33) had the lowest; Family Factors averaged 13.63 ± 3.17. Institutional and policy support were therefore perceived more strongly than employment- and internet-related pressures. Gender-based comparisons showed no statistically significant difference in any of the six influencing dimensions (all *p* ≥ 0.197). The largest numerical difference was observed in peer relationships, where female students scored 0.53 points higher on average (t = −1.30, *p* = 0.197, d = 0.29) (Table 4).

**Table 4:** Gender differences in influencing factors (*N* = 123).

Dimension	Male (M ± SD)	Female (M ± SD)	t	*p*	Cohen’s d
Family factors	13.42 ± 3.29	13.69 ± 3.15	−0.38	0.704	0.08
School education	15.54 ± 2.77	15.62 ± 3.63	−0.10	0.917	0.02
Online ecology	13.23 ± 3.44	13.24 ± 3.32	−0.01	0.993	0.00
Peer relationships	13.65 ± 1.81	14.18 ± 1.82	−1.30	0.197	0.29
Employment pressure	12.38 ± 1.92	13.05 ± 2.48	−1.27	0.206	0.28
Policy perception	14.46 ± 2.61	14.88 ± 3.21	−0.61	0.545	0.13

### 3.6 Grade-level differences in influencing factors

No statistically significant difference across academic years was observed for any of the six influencing dimensions (all *p* ≥ 0.158). The largest between-year effect was seen for school education (*F* = 1.76, *p* = 0.158, η^2^ = 0.043), where first-year students recorded the highest score (16.59 ± 3.15) and third-year students the lowest (14.92 ± 4.16); the remaining dimensions showed only small and non-significant variation (Table 5). As with the dimension scores, these comparisons rest on a sample that can detect only medium-to-large between-group effects.

**Table 5:** Differences in influencing factors by academic year (*N* = 123).

Dimension	Year 1	Year 2	Year 3	Year 4	*F*	*p*	η^2^
Family factors	13.22 ± 2.85	13.68 ± 2.98	13.59 ± 3.54	14.61 ± 3.33	0.80	0.494	0.020
School education	16.59 ± 3.15	15.28 ± 3.05	14.92 ± 4.16	15.28 ± 2.67	1.76	0.158	0.043
Online ecology	12.46 ± 3.01	13.52 ± 3.29	13.51 ± 3.71	14.00 ± 3.18	1.21	0.311	0.029
Peer relationships	14.39 ± 1.64	13.64 ± 1.66	14.05 ± 2.09	13.94 ± 1.83	0.91	0.437	0.022
Employment pressure	13.22 ± 2.12	12.80 ± 2.35	12.85 ± 2.74	12.50 ± 2.28	0.43	0.732	0.011
Policy perception	15.05 ± 2.84	14.76 ± 2.42	14.49 ± 3.86	14.89 ± 2.76	0.22	0.880	0.006

### 3.7 Regression analysis of influencing factors

The six influencing factors were entered simultaneously as predictors of the observed composite positive-social-mentality index in a standardized multiple regression (Section 2.4); the resulting coefficients are reported in Table S2. The measurement model on which the composite scores rest is described in Section 3.1. Because the estimates are computed on observed composite scores rather than fitted through a latent-variable measurement model, they are reported as standardized regression coefficients (β) and are subject to the caveat set out in Section 4.7. Results revealed:

School education (β = 0.68, *p* < 0.001) was by far the strongest positive predictor of social mentality, and peer relationships (β = 0.18, *p* < 0.001) and family factors (β = 0.17, *p* = 0.007) were also positively associated with it.
Policy perception (β = 0.16, *p* = 0.019) showed a positive, though weaker, association, while online ecology (β = −0.14, *p* = 0.007) was negatively associated with social mentality.
Employment pressure (β = −0.09, *p* = 0.140) was not a significant predictor once the other five factors were controlled.

The six factors together explained 81% of the variance in social mentality (R^2^ = 0.81).

The exploratory mediation analysis described in Section 2.4, estimated on the same observed composite scores, further revealed that peer relationships partially mediated the relationship between school education and social mentality (indirect effect: β = 0.09, *p* = 0.004).

## 4 Discussion

### 4.1 Summary of findings

This study provides empirical insights into the correlates and characteristics of positive social mentality among college students in the context of the new era. The findings confirm that the majority of students demonstrated an optimistic outlook toward the future, with value identity being the strongest dimension and growth mindset the weakest. No significant differences were found by gender or academic year, and the regression model identified school education as by far the strongest positive correlate, with smaller positive contributions from peer relationships, family factors and policy perception, and a negative association with online ecology.

### 4.2 Theoretical implications

The findings align with and extend multiple theoretical frameworks:

Bandura’s self-efficacy theory (1977, 1997): Self-efficacy was rated slightly above the overall index in this sample (15.51 ± 3.16 versus 15.34), which is consistent with the view that mastery experiences are an important source of confidence. The expected grade-level accumulation of mastery experiences was not, however, reflected in the present data: self-efficacy did not differ significantly across academic years (*F* = 0.90, *p* = 0.444), and in a cross-sectional sample mastery accumulation cannot be observed directly. The theoretical account therefore remains plausible but is not tested here.
Bronfenbrenner’s ecological systems theory (1979): The significant contributions of family factors (microsystem), school education (mesosystem), and policy perception (exosystem) to social mentality demonstrate how multiple environmental layers interact to shape psychological outcomes. The finding that peer relationships partially mediate the school education–social mentality relationship further highlights the importance of proximal processes in development.
Positive psychology (Seligman and Csikszentmihalyi, 2000): The multidimensional structure of social mentality identified in this study—encompassing optimism, resilience, self-efficacy, and social responsibility—aligns with the PERMA model’s emphasis on positive emotions, engagement, relationships, meaning, and accomplishment. The relatively weaker scores in growth mindset, psychological resilience and emotional regulation (Table 1) suggest that these are critical areas for intervention, consistent with the positive psychology emphasis on building emotional and motivational competencies.
Social mentality theory (Wang and Yang, 2018; Wang, 2020): The strong endorsement of value identity and social responsibility among participants reflects the collective-oriented values emphasized in Chinese society. This finding underscores the cultural embeddedness of social mentality and the importance of considering socio-cultural context in understanding its formation.

### 4.3 Interpretation of key findings

Gender differences: No dimension showed a statistically significant gender difference; the largest numerical gap was 0.81 points on value identity (d = 0.28), and every other dimension differed by less than one third of a standard deviation (Table 2). The absence of a gender effect is itself informative: the gendered differences in emotional sensitivity, social support-seeking and internalized responsibility reported elsewhere (Fredrickson, 2001; Taylor et al., 2000) were not reproduced in this sample. With 26 male and 97 female respondents the study could detect only standardized differences of d ≥ 0.62 at 80% power (Section 2.1), so the present data are consistent with no difference but cannot exclude a small-to-moderate one.

Grade-level trajectory: The expected decline from the first to the third year, followed by partial recovery in the fourth, was not supported: no dimension differed significantly across academic years and no monotonic trend was present (Table 3). The numerical pattern is nevertheless of interest - first-year students recorded the highest overall index (15.94 ± 2.16), the third-year group the lowest (15.16 ± 3.11), and value identity and sense of belonging were highest in the first year - but with N = 123 across four groups the study could detect only η^2^ ≥ 0.08 (Section 2.1), so these differences are best regarded as inconclusive. Whether the trajectory described in the literature reflects a genuine developmental process will require a larger, longitudinal sample.

Socioeconomic context: Family economic status was recorded as a demographic variable (Appendix, Part I), but the present study did not carry out a stratified analysis by economic background, and no socioeconomic finding is reported here. The literature indicates two competing possibilities for this population—that economic disadvantage may constrain confidence through resource disparity, or that moderate adversity may foster resilience through a steeling effect (Seery, 2011)—and distinguishing between them requires a larger, stratified sample containing sufficient numbers of students in each economic category. We accordingly identify the socioeconomic gradient of positive social mentality as a priority for future work rather than as a finding of the present study.

### 4.4 Alternative explanations and moderators

Several alternative explanations warrant consideration. Because no gender difference reached significance, the gendered patterns reported in the literature are not reproduced here, and the small observed differences lie within the range that this sample cannot resolve. Likewise, the grade-level pattern was not statistically reliable, and individual differences in coping strategies - active copers showing less decline than avoidant copers - remain a plausible moderator that a larger sample would be needed to test. A third candidate is social support, which buffers against stress and enhances positive adaptation (Cohen and Wills, 1985); whether peer and family support operate in this way in the present sample cannot be determined from cross-sectional data alone.

### 4.5 Cross-cultural considerations

The cultural context of this study is important to consider. China’s collectivist values, emphasis on social harmony, and the institutional promotion of core socialist values create a unique environment in which social mentality develops. The strong endorsement of value identity and social responsibility in this study likely reflects these cultural norms. Findings may not generalize to Western or individualist cultural contexts, where autonomy and personal achievement may be more emphasized (Hofstede, 2001). Future cross-cultural validation studies are needed to determine the universality versus cultural specificity of the observed patterns.

### 4.6 Practical recommendations

Based on our findings, we propose a three-tier intervention strategy:

Tier 1 – Universal interventions (for all students):

- Integrate positive psychology and resilience training into the core curriculum
- Promote a healthy campus culture through inclusive extracurricular activities
- Implement regular mental health screenings using validated instruments (e.g., GSES)
- Enhance digital literacy programs to mitigate negative online influences

Tier 2 – Selective interventions (for at-risk groups):

- Early Clinical Immersion Program for first-year students: Introduce standardized patient simulation training and phased micro-skill operation certification to help students accumulate early success experiences.
- Urban–Rural Mentorship System: Establish partnerships between affiliated hospitals and primary care units to provide one-on-one guidance and resource sharing for students from rural areas.
- Campus Belonging Initiative: Develop peer mentoring programs and community-building activities specifically for upper-year students to combat declining sense of belonging.

Tier 3 – Indicated interventions (for individuals with identified needs):

- Individual counseling and psychological support for students with low social mentality scores
- Career counseling to address employment pressure anxiety

Implementation monitoring: We recommend incorporating the GSES into annual psychological assessments for medical students, establishing individual efficacy development files for each student to achieve long-term tracking and precise intervention.

### 4.7 Limitations and future directions

Several limitations should be acknowledged:

Sample constraints: The sample was limited to a single institution, a single major (medical laboratory science), and a relatively small size (N = 123). The gender ratio (78.86% female), while consistent with the demographic composition of the field in China, limits gender-based generalizability. All participants were of Han ethnicity, which reflects the local demographic context but precludes analysis of ethnic minority perspectives. These constraints mean that findings are preliminary and may not generalize to other disciplines, institutions, or regions (Henrich et al., 2010). In addition, with N = 123 and six predictors the regression model reported in Section 3.7 rests on a modest number of observations, and it was specified on observed composite scores rather than through a latent-variable measurement model. The coefficients in Table S2 are therefore standardized regression coefficients rather than structural-equation-model path estimates; they take no account of measurement error in either the predictors or the outcome, and they should be regarded as preliminary and exploratory rather than definitive. Replication in a substantially larger, multi-centre sample, with the full latent-variable model estimated in dedicated software, is required before these coefficients are treated as stable.
Cross-sectional design: The cross-sectional design and convenience sampling preclude causal inference. Causal relationships cannot be inferred from the observed associations. Longitudinal studies are needed to establish temporal precedence and developmental trajectories.
Self-report bias: The reliance on self-report measures may introduce common method bias and social desirability effects (Podsakoff et al., 2003). While the inclusion of reverse-scored items (n = 4) partially mitigates this, future studies might benefit from incorporating additional reverse-scored items to further enhance measurement validity. The relatively small number of reverse-scored items is a limitation. A further limitation concerns consistency of keying: within four of the six associated-factor categories positively and negatively worded items are combined in the same composite, and only four items in the whole instrument (items 13, 25, 51 and 56) were reverse-coded, so the direction of the composite scores for those categories should be interpreted with caution and future work should adopt a single uniform keying rule.
Measurement scope: The study did not include objective behavioral measures or clinical diagnostic tools. Future research should consider incorporating multi-method assessments including peer ratings, observational measures, and physiological indicators.
Future research directions:

- Multi-center, multi-disciplinary, and more gender-balanced sampling to enhance generalizability
- Longitudinal studies tracking social mentality development across the full medical education trajectory
- Cross-cultural comparative studies to examine cultural specificity versus universality
- Studies intentionally recruiting participants from diverse ethnic backgrounds to enable inter-ethnic comparisons
- Intervention studies evaluating the effectiveness of the proposed strategies
- Qualitative studies exploring the lived experiences underlying quantitative patterns

## 5 Conclusion

This study provides empirical evidence that college students’ positive social mentality is associated with multiple interrelated environmental and relational factors (school education, family factors, peer relationships, online ecology and policy perception). No statistically significant differences were found by gender or academic year, although the numerical pattern was lowest in the third year. School education emerged as by far the strongest positive correlate, with smaller positive contributions from peer relationships, family factors and policy perception, and a negative association with online ecology. These findings highlight the importance of multi-level, targeted interventions that address individual and environmental factors simultaneously. Cultivating a positive social mentality is not only a matter of individual psychological well-being but also a social responsibility that requires concerted efforts from families, educational institutions, policymakers, and communities. By implementing evidence-based, culturally sensitive interventions and investing in supportive environments, we can foster a generation of college students who are not only academically competent but also psychologically resilient and socially engaged—essential qualities for building a harmonious and progressive society in the new era.

## Data Availability

The original contributions presented in the study are included in the article/Supplementary material, further inquiries can be directed to the corresponding author.
