# Supplementary material for: Associated factors and cultivation strategies of positive social mentality among college students in the new era
Source: Front Psychol. 2026 Oct 13;17:1851175. doi: 10.3389/fpsyg.2026.1851175 (PMC13710085; doi:10.3389/fpsyg.2026.1851175)
Supplement: Supplementary file 1 [file Table_1.pdf]

Appendix and Supplementary

Appendix: College Student Positive Social Mentality Questionnaire

Part I: Demographic Information (5 items)

1.Gender: □ Male □ Female

2.Academic Year: □ Year 1 □ Year 2 □ Year 3 □ Year 4

3.Place of Origin: □ Urban □ Rural

4.Political Affiliation: □ CPC Member □ Communist Youth League □ General Public □ Democratic Party

5.Family Economic Status: □ Financial Difficulty □ Average □ Affluent

Part II: Social Mentality Dimensions

Rated on a 5-point Likert scale (1 = Strongly Disagree, 5 = Strongly Agree)

Optimism

6. I believe the future will be better than the present.
7. Even when facing difficulties, I can see the positive side.
8. I am confident in the country's development prospects.
9. I usually expect things to turn out well.

Psychological Resilience

10. I can recover quickly from major setbacks.
11. I remain calm and think clearly under pressure.
12. I can easily adapt to environmental changes.
13. Negative comments from others do not shake my confidence. *(reverse-scored)*

Self-Efficacy

14. I believe I can effectively solve difficult problems.
15. I am confident in my ability to complete new tasks.
16. My decision-making skills are reliable.
17. Even after failure, I reflect and try again.

Social Responsibility

18. I feel obligated to speak up on social issues.
19. Participating in public service makes me feel fulfilled.
20. I actively follow national policy developments.
21. Others' difficulties increase my willingness to help.

Growth Mindset

22. Abilities can be improved through effort.
23. Constructive criticism helps me grow.
24. Challenging tasks excite me more than they cause anxiety.
25. Others' success motivates me rather than makes me jealous. *(reverse-scored)*

Sense of Belonging

26. I feel a strong sense of community at school.
27. Mutual support among classmates makes me feel warm.
28. The campus environment gives me a sense of security.
29. I am willing to contribute for the honor of my class.

Value Identity

30. My values align with the core socialist values.
31. I believe honesty is the foundation of interpersonal relationships.

32. I support fair competition as a way to achieve success.
33. I maintain independent judgment on social issues.

Emotional Regulation

34. I recover quickly from negative emotions.
35. I actively seek ways to relieve stress.
36. I rarely remain upset over minor issues for long.
37. I maintain a healthy balance between study and recreation.

Part III: Influencing Factors (6 categories × 4 items = 24 items)

Rated on a 5-point Likert scale (1 = Strongly Disagree, 5 = Strongly Agree)

Family Factors

38. My parents often discuss life values with me.
39. Family financial stress significantly affects my psychological state.
40. My family's expectations give me positive motivation.
41. Family conflict diminishes my confidence in interpersonal relationships.

School Education

42. My ideological and political teachers resonate with me.
43. My counselor's care gives me a sense of support.
44. Campus cultural activities enhance my group identity.
45. Value guidance in professional courses has a deep impact on me.

Online Ecology

46. Negative information on social media lowers my social trust.
47. Online violence affects my sense of interpersonal safety.
48. Positive short videos improve my emotional state.
49. Controversial online debates confuse my values.

Peer Relationships

50. My friends' support gives me courage in adversity.
51. Competition among classmates makes me anxious. *(reverse-scored)*
52. Dormitory relationships affect my emotional stability.
53. I'm willing to confide my mental struggles to close friends.

Employment Pressure

54. Job market competition affects my confidence in the future.
55. Internship experience makes me more optimistic about career prospects.
56. I worry about the employment outlook for my major. *(reverse-scored)*
57. Innovation and entrepreneurship policies ease my employment anxiety.

Policy Perception

58. Rural revitalization policies enhance my confidence in social equity.
59. The "Double Reduction" policy influences my career planning.
60. COVID-19 control measures make me appreciate institutional strengths.
61. Social security policies reduce my life-related anxieties.

Scoring Guidelines

1.**Positively Worded Items**

Scored directly using the original Likert scale:

1 = Strongly Disagree

2 = Disagree

3 = Neutral
4 = Agree
5 = Strongly Agree
2.**Reverse-Scored Items**
Items 13, 25, 51, and 56 are reverse-coded using the formula:
Score = 6 − Original Score
(e.g., an original score of 1 is converted to 5)
3.**Dimension Scores**
Each dimension consists of four items.
Dimension Total = Sum of 4 Items (Range: 4–20)
4.**Overall Social Mentality Index**

Calculated as the unweighted mean of the eight dimension scores, each dimension carrying an equal weight of 1/8 (0.125).**Index = (Σ Dimension Score) / 8**

Supplementary Materials

Table S1. Measurement Properties of the Eight Dimensions and Their Weighting

**Dimension**	**Factor Loadings**	**Composite Reliability (CR)**	**AVE**	**Weight (equal)**
Optimism	0.72-0.88	0.91	0.73	0.125
Psychological Resilience	0.68-0.85	0.89	0.69	0.125
Self-Efficacy	0.74-0.91	0.93	0.77	0.125
Social Responsibility	0.63-0.79	0.86	0.61	0.125
Growth Mindset	0.70-0.83	0.88	0.65	0.125
Sense of Belonging	0.67-0.82	0.85	0.60	0.125
Value Identity	0.71-0.86	0.90	0.70	0.125
Emotional Regulation	0.66-0.84	0.87	0.63	0.125

Note: The factor loadings, composite reliability and AVE values are those reported for the confirmatory factor analysis of the eight dimensions. The overall social mentality index is,

however, an unweighted mean of the eight dimension scores (Section 2.2.2), so every dimension carries an equal weight of 0.125 and the weights sum to 1.00. No regression- or SEM-derived coefficient is applied to the index; the weight values previously printed in this table summed to 1.18 and were retained only as originally reported, so they have been replaced by the equal weights actually applied.

Table S2. Standardized Regression Coefficients of the Six Influencing Factors

Factor	Standardized Coefficient (β)	*p* Value	Semi-partial R^2
Family Factors	0.17	0.007	0.01
School Education	0.68	<0.001	0.16
Online Ecology	-0.14	0.007	0.01
Peer Relationships	0.18	<0.001	0.02
Employment Pressure	-0.09	0.140	0.00
Policy Perception	0.16	0.019	0.01

Interpretation:

Positive predictors: School education (β = 0.68) is by far the strongest positive contributor to a positive social mentality, followed by peer relationships (β = 0.18), family factors (β = 0.17) and policy perception (β = 0.16).

Negative predictor: Online ecology (β = –0.14, p = 0.007) is negatively associated with a positive social mentality, whereas employment pressure (β = –0.09, p = 0.140) is not a significant predictor. The six factors together explain 81% of the variance ($R^2 = 0.81$).

Interpretation caveat: The coefficients are standardized regression coefficients estimated on observed composite scores, not latent-variable structural-equation-model path estimates (Section 4.7); the variance column contains semi-partial R^2 values, whose sum (0.21) is not the model R^2 (0.81, Section 3.7).
